# Anatomical Variations in Clefts of the Lip with or without Cleft Palate

**DOI:** 10.1155/2012/542078

**Published:** 2012-11-29

**Authors:** K. Carroll, P. A. Mossey

**Affiliations:** Dental Hospital and School, University of Dundee, 1 Park Place, Dundee DD1 4HR, UK

## Abstract

*Objective*. Few orofacial cleft (OFC) studies have examined the severity of clefts of the lip or palate. This study examined associations between the severity of cleft of the lip with cleft type, laterality, and sex in four regional British Isles cleft registers whilst also looking for regional variations. *Design*. Retrospective analysis of cleft classification in the data contained in these four cleft registers. *Sample*. Three thousand and twelve patients from cleft registers based in Scotland, East England, Merseyside, and Belfast were sourced from the period 2002–2010. Submucous clefts and syndromic clefts were included whilst stillbirths, abortuses, and atypical orofacial clefts were excluded. *Results*. A cleft of the lip in CLP patients is more likely to be complete in males. A cleft of the lip in isolated CL patients is more likely to be complete in females. Variation in the proportion of cleft types was evident between Scotland and East England. *Conclusions*. Association between severity of cleft of the lip and sex was found in this study with females having a significantly greater proportion of more severe clefts of the lip (CL) and CLP males being more severe (*P* < 0.0003). This finding supports a fundamental difference between cleft aetiology between CL and CLP.

## 1. Introduction

Maintaining a register of children born with orofacial clefts is recognised as important with regards to audit, research, and the planning and provision of services [1]. The use of a simple classification, such as the LAHSAL system proposed by Kriens in 1989 [2] to describe clefts is recognised as being of prime importance and allows for the accurate recording of cleft types and comparison between locations [3].

The evidence available at a global and European level indicates very significant regional variation in the birth prevalence of orofacial clefts, both cleft palate (CP) and cleft lip and palate (CLP) [4]. It is well known that the aetiology of orofacial clefts is characterised by heterogeneity and that the aetiology is polygenic multifactorial with both environmental and genetic factors contributing to nonsyndromic type [5], which comprises approximately 70% of all orofacial clefts.

The increase in CP seen in some UK studies and in parts of Scandinavia may be as a result of factors associated with their northern position [6]. The proportion of CP in Sweden was shown to increase with the increase in latitude at which the comparison was carried out [7].

Several studies have shown that females are affected more often than males with regards to isolated CP [6, 8, 9]. Conversely, a predilection for the male sex is observed in clefts involving the lip [9, 10].

With regards to laterality, most studies show a left-sided dominance of clefts involving the lip [11–13]. Contrasting results have been obtained upon examining for a link between sex and laterality. In South-East Scotland, a male dominance for left-sided clefts was recorded [14]. Despite an earlier report in Northern Ireland of a predilection for right-sided clefting in females, a subsequent analysis over a twenty-year period failed to find an association between sex and laterality [15].

Animal studies have shown that during development, the left palatal shelf takes longer to rotate into the horizontal position leaving this side susceptible to developmental interruption for longer [16]. A suggested reason for this is the lower arterial pressure on the left side compared to the internal carotid artery on the right side [13]. Conversely with regard to all other external congenital anomalies, an excess on the right side has been noted [17].

A possible hypothesis for this is revealed in rat embryos where the mitochondrial maturation rate is delayed on the right side making this side more susceptible to prenatal hypoxia [17]. Although this right-sided correlation for most congenital anomalies is not replicated in orofacial clefts, the author goes on to explain that male sex hormones lower mitochondrial respiration rates which could help to explain the male predominance of CL.

Very significant progress has been made in recent years with respect to genetic determinants and a range of environmental risk factors [18]. Current research has resultantly focussed on finding out more about the interactions between genes and environment, the influence of epigenetics, and the targeting of environmental factors that in the presence of genetic polymorphisms become teratogenic.

Research in this field in the past has been hampered by individual population studies, small sample sizes, pooling of a range of different cleft phenotypes in a single analysis, and potentially masking any differences between different subphenotypes of clefts.

One aim of this study was to profile the details of cleft lip ± palate patients from four British Isles cleft registers, recorded in accordance with the LAHSAL system. The majority of papers that have profiled cleft registers do so with regard to the relative proportions of cleft type, sex and laterality. Emphasis has recently been placed on elucidating further subphenotypes associated with orofacial clefts and determining characteristics such as heritability and transmission patterns of orofacial cleft subphenotypes [18]. Research has been carried out to determine further subphenotypes of clefts, including parental features, in order to help unravel the genetic basis for the condition [19].

A further aim of this study was to analyse the registers with regards to the severity of the cleft of the lip and possible associations with laterality and sex. Until recently, the importance of the severity of the cleft of the lip was described only in relation to the optimum timing of surgery and the surgical technique involved [20]. Criticism has recently been apportioned to the recording of cleft type by its presence or absence as being too simplistic which may hamper the genetic determination of orofacial clefts [18]. The identification of subphenotypes within cleft lip could aid recurrence risk estimations and help to refine gene mapping. The profiling of the “severity of cleft” subphenotype in conjunction with the other main variables may present a finding that is relevant as an expression of the genetic and environmental factors underpinning clefts.

## 2. Method

A retrospective study was undertaken to identify all children born with a cleft of the lip ± cleft palate in the areas covered by the cleft registers in Scotland, East England, Belfast, and Mersey during the period January 2002 to April 2010. The criteria for entering clefts onto the databases were similar in all 4 regions in that abortuses, stillbirths, and atypical orofacial clefts were excluded. Otherwise all typical clefts of the lip and palate were placed on the register, whether or not they were diagnosed at that point with a syndrome, and submucous clefts if detected were also included.

The purpose of selecting these geographical locations was due to the reported differences in prevalence of the various forms of cleft in these areas. Studies report a majority of cleft palate cases in Scotland [5, 13, 21] and Northern Ireland [8].

The databases were downloaded at the commencement of the study in January 2010. The aim was to record information from anonymised data relating to the cleft type, date of birth, sex, side affected, and the severity of the cleft. The registers were compared by interpreting the LAHSAL code assigned to each patient registered with a cleft, where the letters of LAHSAL represent the two sides of the lip and alveolus (the first L and A indicate the right lip and alveolus) and the hard and soft palate. Upper and lower case letters are used to depict “complete” and “incomplete” clefts.

The definition of complete cleft is an area of contrasting explanations in the literature. For the purpose of this study, “complete cleft” relates to a cleft which involves the full height of the lip to the nasal sill (and therefore is the most severe type), whereas “incomplete cleft” relates to those which only involve a portion of the height of the lip (and is less severe). Many articles refer to a complete cleft as one which communicates between the lip and the palate, that is, CLP.

Whilst bilateral cases were included in the totals of clefts for each region, they were excluded from the analysis examining for a link between cleft type and severity, on the basis of the need to record one type of phenotype per case i.e. some bilateral cleft patients had one complete cleft on one side and an incomplete cleft on the contralateral aspect.

## 3. Results

**Figure 1:**
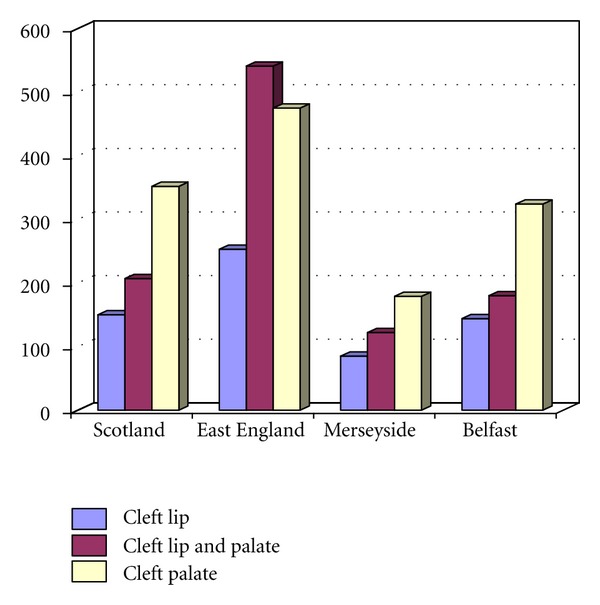
The distribution of different types of OFC across 4 UK regions.

In this retrospective comparative analysis of cleft classification of populations contained in four British Isles cleft registers, a total of 3012 patients from cleft registers based in Scotland, East England, Mersey, and Belfast were sourced from the period 2002 to 2010. The number of patients in each category is indicated in the Figure 1 and Tables 1, 2, 3, and 4.

## 4. Discussion

### 4.1. Cleft Type Proportions in Scotland and East England

The proportion of cleft palate as a percentage of all clefts in Scotland was 50%. The proportion for CLP was 29%. This finding is in keeping with previous results from the west of Scotland [21] which showed a predominance of cleft palate at 52%. The result for CP is also similar to the 53% figure obtained in N. Ireland [15] which is nearby geographically and could be said to have a similar population to Scotland in the genetic sense. The proportion of CP detected in East England was 43%. This is in keeping with the lower prevalence of CP detected in previous English studies in Birmingham [22], Northumberland [9], and the Trent region [23] which recorded CP at 40%, 33%, and 39%, respectively.

The prevalence of CLP in Scotland in this study at 29% again compares favourably with the results gained from the west of Scotland [21] in 1987 (34%) and N. Ireland [8] in 1994 (30%). The prevalence obtained from East England in this study was 37%. The same figure of 37% was obtained from Birmingham [22] in 1953 and 36% from Northumberland [9] in 1962. In 1988, Trent region [23] recorded a combined figure for CL and CLP of 61%. The corresponding figure from this study was 57%.

The percentages of cleft type, sex and laterality proportions were found to be very similar between the datasets from Scotland, Merseyside, and Belfast.

### 4.2. Severity of Cleft—Association with Cleft Type

In the four registers examined, the cleft in unilateral CL was found to be predominantly incomplete—69%. The opposite was true with unilateral CLP where the cleft of the lip tended to be complete—88%.

Only two previous studies describing the complete versus incomplete proportions of clefts of the lip and palate have been found in the literature in Brazil and Norway. In the Brazilian study from a cleft and craniofacial centre in Bauru, the majority of unilateral cleft lip cases were incomplete [24], while the Norwegian study reported that for CL, 18% were complete clefts of the lip and primary palate, and for CLP 81% of the lips were complete [25]. This represents consistency in the association between cleft type and the severity of the cleft of the lip. Severity of the cleft of the lip in unilateral CLP was not described in the Brazilian study.

Several studies have shown that comparing congenital anomaly data from different locations can reveal variable characteristics and proportions. A difference in the proportion of cleft types is reported upon in Glasgow in comparison to other locales with the suggestion that this may be due to the interaction of an unidentified environmental teratogen with a susceptible population [21].

An epidemiological study in the UK has shown that true regional variation exists in the prevalence of specific congenital anomalies such as neural tube defects (NTDs), diaphragmatic hernia, and gastroschisis with higher prevalence rates in northern regions such as Glasgow and the north of England [26].

No association could be found regarding the severity of the cleft of the lip and the sex of the patient. Males and females both had a statistically significant level of complete cleft lip in unilateral CLP. While cleft lip is consistently more frequent on the left side, the laterality of the cleft was not associated with the severity of the cleft in either this UK study and the report from Norway. In the Norwegian study it was also reported that in bilateral cleft lip severity was similar on both right and left sides.

### 4.3. Severity of Cleft—Association with Sex

Upon combining the data from the four British Isles registers, complete cleft of the lip in CLP patients was found to occur in 90% of males and 85% of females. In isolated CL patients, complete cleft of the lip occurred in 39% of females and 25% of males. Logistic regression analysis of the data revealed that the differences in proportion of complete and incomplete clefts between males and females for these two groups of patients (CL and CLP) were significant (*P* < 0.0003, *χ* ^2^ = 13.23).

When CL and CLP patients were considered as one entity, that is, CL ± P, no association was found between severity of cleft and the sex of the patient (*P* < 0.356, *χ* ^2^ = 0.852) or between the severity of cleft and the side affected (*P* < 0.530, *χ* ^2^ = 0.394).

This study utilised datasets which excluded atypical lip and facial clefts but did not specifically exclude syndromes which included orofacial clefting as part of the phenotype. The majority of syndromic cleft cases involve patients where the cleft is of the palate only. For example, in Scotland 67% of cases of syndromic clefts where the cleft type was described involved cleft of the palate. This study was concerned with severity of clefts of the lip, and there is evidence of overlap between syndromic and nonsyndromic clefts in terms of aetiology, so for the purposes of this study it was considered acceptable to retain the full data set for the severity analyses.

### 4.4. The Multifactorial Threshold Model

The multifactorial threshold (MFT) model is often used to describe the aetiology of orofacial clefting, that is, no one single causative factor accounts for the development of orofacial clefting.

The MFT model does apply to the data in this study when examining the severity of cleft data for cleft lip and palate (CLP) patients. More males than females are affected by CLP as would be predicted by the MFT model; this study shows that more males have a complete cleft of the lip than females. However, for CL the results of this study do not appear to be compatible with the MFT model of aetiology when considering gender and severity combined. The MFT model for isolated cleft lip (CL) would predict more males to be affected by the more severe (complete) cleft of the lip than females. However, the opposite is observed in CL patients, in that females are affected by a complete cleft of the lip more often than males. This points to a different mechanism for the cause or predisposition to CL as opposed to CLP, supporting previous epidemiological findings [27–29] and genetic evidence [30], and thus providing further circumstantial evidence for a different genetic mechanism in these 2 cleft subphenotypes.

## 5. Conclusion

Cleft lip and cleft lip and palate have traditionally been grouped together epidemiologically as cleft lip ± palate (CL(P)) and considered as one genetic entity in separation from isolated cleft palate (CP), and this has undoubtedly hampered genetic investigations. Based upon the findings in this study relating to severity of cleft and sex, this study provides further evidence that cleft lip may be a distinct genetic entity to cleft lip and palate.

This UK-based study reveals that substantial variation in the proportion of cleft types was evident between Scotland and East England. Furthermore among CLP patients, the cleft of the lip is more severe in males, while the cleft of the lip in isolated CL patients is more severe in females. This association between severity of cleft of the lip and sex was statistically significant (*P* < 0.0003) and supports a fundamental difference between cleft aetiology between CL and CLP.

Further studies are required to determine proportions of cleft severity subphenotypes from centres around the world and to examine possible association with aetiology; more work is also required to standardise and validate the codes in cleft registers to ensure the accuracy and consistency of recording by referring to original clinical photographs and models.

## Figures and Tables

**Table 1:** Cleft types in all four UK regions.

	Cleft lip	Cleft lip and palate	Cleft palate
Scotland	150	207	352
East England	253	541	475
Merseyside	85	122	179
Belfast	144	180	324

**Table 2:** Cleft lip—severity of cleft according to gender and laterality.

	Scotland		Cambridge		Belfast		Liverpool	
	*C *	*I *	*C *	*I *	*C *	*I *	*C *	*I *
Overall	38	95	72	137	39	87	23	53
Males	19	60	36	94	20	57	15	37
Females	19	35	36	43	19	30	8	15
Right side	11	38	24	46	10	35	13	14
Left side	27	57	48	91	29	52	11	38
Males—left	11	34	26	61	13	31	4	28
Females—left	16	23	22	30	16	21	5	10
Males—right	8	26	10	33	7	26	11	9
Females—right	3	12	14	13	3	9	3	5

These figures for CL reveal a reasonable level of consistency across regions in proportions of complete to incomplete clefts with incomplete clefts being consistently more prevalent than complete ones. Also complete clefts of the lip seem to occur more frequently in females. Bilateral clefts were excluded from this analysis.

**Table 3:** Cleft lip and palate—severity of cleft according to gender and laterality.

	Scotland		Cambridge		Belfast		Liverpool	
	*C *	*I *	*C *	*I *	*C *	*I *	*C *	*I *
Overall	125	20	273	35	111	17	66	8
Males	81	11	169	17	73	9	55	7
Females	44	9	104	17	38	8	22	2
Right side	52	5	88	16	47	5	23	4
Left side	73	15	185	19	64	12	53	7
Males—left	45	8	115	9	42	6	37	5
Females—left	28	7	70	9	22	6	16	1
Males—right	36	3	54	8	31	3	17	2
Females—right	16	2	34	8	16	2	6	1

These figures for CLP reveal a reasonable level of consistency across regions in proportions of complete to incomplete clefts with complete clefts being consistently more prevalent than incomplete clefts. Bilateral clefts were excluded from this analysis.

**Table:** (a)

	Male Cleft lip Incomplete	Female Cleft lip Incomplete	Male Cleft lip Complete	Female Cleft lip Complete
Scotland	60	35	19	19
Cambridge	94	43	36	36
Belfast	57	30	20	19
Liverpool	37	15	15	8

**Table:** (b)

	Male Cleft lip and palate Incomplete	Female Cleft lip and palate Incomplete	Male Cleft lip and palate Complete	Female Cleft lip and palate Complete
Scotland	11	9	81	44
Cambridge	17	17	169	104
Belfast	9	8	73	38
Liverpool	7	2	55	22

These data reveal that the most common single cleft subphenotype in every region in the UK is a complete cleft of the lip on the left side in a male, with an accompanying cleft of the palate. Bilateral clefts were excluded from this analysis.
